# Knowledge, attitudes and practices towards rabies prevention among residents of Abuja municipal area council, Federal Capital Territory, Nigeria

**DOI:** 10.11604/pamj.2018.31.21.15120

**Published:** 2018-09-10

**Authors:** Aboyowa Arayuwa Edukugho, Jarlath Udoudo Umoh, Matthew Diem, Oyetunji Ajani, Belinda Uba, Lilian Okeke, Elizabeth Adedire, Adewole Adefisoye, Caroline Edukugho, Patrick Nguku

**Affiliations:** 1 Nigeria Field Epidemiology and Laboratory Training Program, Abuja, Nigeria; 2 Ahmadu Bello University, Zaria, Kaduna State, Nigeria

**Keywords:** Knowledge, attitude, practice, rabies, dog-bite

## Abstract

**Introduction:**

Rabies is a fatal neglected zoonosis killing 55,000 people worldwide annually. It is endemic in Nigeria with 10,000 people exposed annually through dog-bites. To inform adequate intervention due to the rise in reported dog-bite cases, we assessed knowledge, attitudes and practices (KAP).

**Methods:**

We carried out a cross-sectional study among 224 residents in Abuja Municipal Area Council. We used structured questionnaires to collect data on sociodemographic characteristics, knowledge and attitudes on rabies prevention. Knowledge score of ≥ 8 points based on ten-point domain question were regarded as satisfactory. We analyzed data using Epi-Info and Excel for proportions and associations were determined at 5% significance level.

**Results:**

A total of 123 (55%) respondents owned dogs. One hundred and eighty four (82%) respondents had satisfactory knowledge on rabies prevention, about 87% knew where to get dogs vaccinated and would seek medical treatment from the hospital when bitten by a dog. Majority (58%) did not know the frequency of anti-rabies administration for dogs and 63.3% did not know the appropriate first aid actions following dog-bites. Of 123 dog owners, 35% would allow their dogs roam without restriction and 94% reported vaccinating their dogs against rabies. Dog owners were more likely to have satisfactory knowledge of rabies compared to non-dog owners (OR 7.8, 95% CI 1.0-62.0, p = 0.02).

**Conclusion:**

Knowledge on rabies is satisfactory but with gaps in the frequency of dog anti-rabies vaccination, appropriate first-aid following dog bites and non restriction of dog movement. To prevent rabies, these gaps need to be addressed through public enlightenment and enforcement of dog movement restrictions laws.

## Introduction

Rabies is a widely distributed viral zoonotic disease of major public health importance that affects human, domestic and wild animals. It remains incurable and survivors are extremely rare [1]. More than 3 billion people globally are living in countries/territories where dog rabies still exists and are potentially exposed to the disease. In many countries of those continents, few activities are underway to prevent rabies occurrence in humans and to control rabies in dogs, even when the number of human deaths is high [2]. Dog licensing, killing of stray dogs, muzzling, and other measures contributed to the elimination of rabies from the United Kingdom in the early 20^th^ century. More recently, large-scale vaccination of cats, dogs and ferrets has been successful in combating rabies in many developed countries [2]. Almost all human deaths caused by rabies occur in Asia and Africa. It is estimated that at least 55,000 human rabies deaths occur yearly following contact with rabid dogs. Developing countries with a large number of dogs, most of which movement are not restricted, account for approximately 98 percent of human rabies [2]. The first documentation of rabies in Nigeria in humans was in 1912 and in dog in 1925 [3] and since then many authors [4-8] have established that the disease is endemic in the country with more recent reports [2] showing that the prevalence of the disease is on the increase. The dog is the principal host that maintains and transmits the disease to other animals and man in Nigeria [9-13] where an estimated 10,000 people are exposed annually through dog-bites. Undocumented reports received by the Federal Department of Livestock and Pest Control Services in the Federal Ministry of Agriculture and Rural Development, Abuja, Nigeria seem to suggest an increase in the case of dog bite in the Federal Capital Territory (FCT) Abuja, Nigeria in the last five years. Transmission of rabies to human can be greatly reduced by health information and behaviour of humans towards the disease. Therefore, information on perception of rabies by the residents of FCT has the benefits of enabling the health agencies to better evaluate and plan for a more efficient and effective rabies control program. This study assessed the knowledge, attitudes and practices of the residents of Abuja Municipal Area Council (AMAC) towards rabies.

## Methods

**Study area:** This study was conducted in Abuja Municipal Area Council (AMAC) which is one of the six (6) Area councils in FCT Abuja, Nigeria with the population size of 776,298 [14]. It is a well-planned city that is multi-cultural, cosmopolitan and one of the wealthiest cities in Africa. There are 37 registered veterinary practicing premises out of which 2 are government owned while the remaining 35 are privately owned. Health facilities owned by government and privately-owned are spread across the Area Council. These are managed by registered veterinary and health professionals who can adequately respond to and manage cases of dog bites.

**Study design:** This was a descriptive cross-sectional study.

**Study population:** Residents of AMAC who were 18 years and above who gave their consent were eligible to participate.

**Sample size:** A convenient minimum sample size of 224 was used for the study.

**Sampling technique:** A multistage sampling was employed. Three districts were chosen out of the nine districts through simple random sampling. One street in each district was also randomly picked. The first household was randomly picked, and every other household was selected systematically until the required number was achieved. An eligible member of the household was selected though balloting.

**Study instruments:** A semi-structured interviewer administered questionnaire with the following sections: socio-demographic, knowledge, attitude and practice was employed.

**Data collection methods:** The questionnaire was pre-tested in one of the districts in the study area that was not selected for the study. The questionnaires were then analyzed and changes were made based on the feedback received. Thereafter, two trained interviewers were assigned to each of the selected districts to administer the questionnaire. The first section sought socio-demographic data while the other three sections sought information on the knowledge, attitudes and practices towards rabies prevention. The variables; knowledge, attitudes and practices were graded on a scale into ten domains, a point was scored for correct responses and zero for wrong responses with a maximum score of ten and a minimum score of zero. Total score of 0-4 was graded as poor, 5-7 as fair while 8-10 as satisfactory.

**Data analysis:** Data was entered, cleaned and analyzed using Microsoft Office Excel and Epi Info TM 3.5.3. Univariate analysis was performed for frequencies, means and proportions of socio-demographic characteristics of respondents and KAP of respondents towards rabies. Chi-square test or Fisher's exact test (2-tailed) was used, as appropriate, to evaluate for significance of differences in responses between dog ownership and knowledge and attitude of respondent towards rabies and association between socio-demographic characteristics and knowledge of respondents. A P-value of <0.05 was considered to be significant.

**Ethical clearance:** Approval was obtained from the Research and Ethics Committee of Ahmadu Bello University, Zaria, Nigeria. Informed consent of the participants in the study were obtained and respondents were assured of confidentiality of information supplied.

## Results

A total of 224 respondents were interviewed. Socio-demographic characteristics of respondents showed that the mean age was 39 ± 5 years and 59.8% were below the age of 40 years. Majority of respondents (62.1%) were males, 75% were married, 94.2% had post-secondary education and 47.8% were on government employment. Fifty five percent owned dogs and 83.5% practiced Christianity as a form of religion (Table 1). Eighty two percent of the respondents had satisfactory knowledge, 18% fair knowledge and none had poor knowledge towards rabies prevention. Respondents attitudes and health seeking behaviour towards rabies prevention showed that 74% had positive attitudes, 24% fair attitudes while only 2% showed negative attitudes. Seventy five percent respondents of the 123 that owned dogs had satisfactory practice, 20% fair practice while 5% had poor practice towards rabies prevention. All the respondents were aware that bite from a rabid dog is the mode of transmission of rabies, that dogs are the main reservoir of rabies in Nigeria, that rabies is fatal, that rabies can lead to death and that rabies can be prevented by vaccination. Approximately 84% respondents knew that rabies affects both humans and animals, 88.8% knew the signs observed in a rabid dog, 87.1% knew where to obtain rabies vaccination for their pets while 42% knew that rabies vaccination is given annually. Most respondents (77.7%) knew that suspected pet head is submitted to the veterinarian, however only 41.5% knew that rabies cannot be cured after symptoms appear (Table 2). About 88% would report a case of dog bite to the local authority, 73.7% would be willing to euthanize pet if found to be rabid while 79.5% would not hesitate to send the head of a suspected rabid animal to the veterinarian. More than 84% of the respondents said the presence of stray dog annoyed them while 75% would support euthanizing stray dogs. Eighty seven percent of respondents would seek medical care in the hospital when bitten by a dog while only 36.6% would apply the appropriate first aid treatment in the event of a dog bite (Table 3). Sixty five percent of dog owners housed their dogs in kennels but sometimes could allow them to freely roam within the compound, 94% patronized veterinary services and had vaccinated their dogs against rabies and other diseases (Table 4). Dog owners were 7.8 times more likely to have satisfactory knowledge of rabies compared to non-dog owners. Single respondents were more likely to have positive attitudes about rabies than the married (Table 5). Dog owners were 2.6 times more likely to be annoyed by the presence of stray dogs than those without dogs (Table 6).

**Table 1:** Frequency distribution of respondents according to socio-demographic characteristics

Variables	Frequency (N)	Percent (%)
Mean age: 39 ± 5 Years		
**Age group (Years)**		
<40	134	59.8
≥40	90	40.2
**Sex**		
Female	85	37.9
Male	139	62.1
**Marital Status**		
Married	168	75.0
Single	56	25.0
**Educational Status**		
Post-Secondary	211	94.2
Secondary	13	5.8
**Type of Employment**		
Private Employment	93	41.5
Government Employment	107	47.8
Self-Employment	24	10.7
**Dog Ownership**		
Own Dog	123	54.9
Do not own Dog	101	45.1
**Religion**		
Christianity	187	83.5
Islam	37	16.5

**Table 2:** Knowledge of respondents towards rabies prevention

Variable	Frequency	Percent (%)
**Host of Rabies**		
Animals	12	5.4
Both	188	83.9
Humans	24	10.7
**Signs of rabies**		
Biting objects and people	199	88.8
Sleeping	6	2.7
Don’t know	19	8.5
**Rabies curable after appearance of symptoms**		
Yes	121	54.0
No	93	41.5
No answer	10	4.5
**Where to obtain rabies vaccination for pets**		
Veterinary clinic	195	87.1
Hospital	18	8.0
Don’t know	11	4.9
**Frequency of administration of rabies vaccination**		
Monthly	15	6.7
Quarterly	14	6.3
Biannually	6	2.7
Annually	95	42.4
Biennially	3	1.3
Don’t know	91	40.6
**Knows that suspected pet head to be submitted to a veterinarian**		
Yes	174	77.7
No	34	15.2
Don’t know	16	7.1

**Table 3:** Attitudes of respondents towards rabies prevention

Variable	Frequency	Percent (%)
**Report dog bite to the authority (veterinarian or police)**		
Yes	196	87.5
No	22	9.8
Don’t know	6	2.7
**Euthanize pet if found to be rabid**		
Yes	165	73.7
No	36	16.1
Don’t know	23	10.3
**Send head of suspected rabid animal to the veterinarian**		
Yes	178	79.5
No	46	20.5
**Annoyed by the presence of stray dogs**		
Yes	190	84.8
No	21	9.4
Sometimes	13	5.8
**Euthanize stray dogs**		
Yes	168	75.0
No	56	25.0
**Seek medical treatment**		
Hospital	26	11.6
Veterinary Clinic	194	86.6
Don’t know	4	1.8
**First aid after dog bite**		
Clean wound with disinfectant	110	49.1
Wash wound with soap and clean running water	82	36.6
Tie wound surface	17	7.6
Don’t know	15	6.7

**Table 4:** Practices of respondents towards rabies prevention

Variables	Frequency	Percent (%)
Keep dog		
Housed in Kennels and free to roam within the compound	80	65.0
Housed in Kennels	23	18.7
Freely roam the compound	12	9.8
No response	8	6.5
**Vaccination against rabies**		
Yes	116	94.3
No	7	5.7
**Other vaccinations**		
Yes	116	94.3
No	7	5.7
**Evidence of vaccination**		
Yes	106	86.2
No	17	13.8

**Table 5:** Association between socio-demographic characteristics and knowledge of rabies

Variables	Knowledge (%)		OR(95%CI)	P value
	Satisfactory	Fair		
**Sex**			0.9(0.4-1.8)	0.81
Male	113(81.3)	26(18.7)		
Female	71(83.5)	14(16.5)		
**Age**				
>40	112(83.6)	22(16.4)	1.3(0.6-2.5)	0.61
≥40	72(80.0)	18(20.0)		
**Marital Status**				
Married	48(85.7)	8(14.3)	1.4(0.6-3.3)	0.55
Single	136(81.0)	32(19.0)		
**Educational Qualification**				
Post-Secondary	11(84.6)	2(15.4)	1.2(0.3-5.7)	0.58
Secondary	173(82.0)	38(18.0)		
**Dog Ownership**				
Own Dog	34(97.1)	1(2.9)	7.8(1.0-62.0)	0.02
Do not own Dog	65(81.3)	15(18.8)		
**Occupation**				
Government Employment	94(87.9)	13(12.1)	2.2(1.1-4.5)	0.05
Private employment	90(76.9)	27(23.1)		

**Table 6:** Association between dog ownership and knowledge and attitudes of respondents towards rabies prevention

Variable	Ownership of Dog		Odds Ratio (95%CI)	P Value
	Own Dog	Do not Own Dog		
	n=123(%)	n=101(%)		
**Knowledge**				
**Frequency of Rabies administration**				
Annual	71(75.5)	23(24.5)	0.2 (0.1-0.4)	<0.001
Others	22(40.0)	78(60.0)		
Attitude				
**Are you annoyed with stray dogs**				
Yes	98(51.6)	92(48.4)	2.6 (1.2-5.9)	0.01
No	25(73.5)	9(26.5)		
**What first aid treatment will you give after dog bite?**				
Wash wound with soap and running water and disinfectant	105(54.7)	87(45.3)	0.9 (0.4-1.9)	0.9
Others	18(56.3)	14(43.7)		

## Discussion

The knowledge among respondents showed an 82% satisfactory knowledge. All respondents showed an acceptable knowledge on the reservoir of rabies, its fatal nature, the signs observed in dogs and its prevention by vaccination. These and knowledge of the administration of rabies vaccine to dogs and what to do with a suspected dog are similar to studies carried out in other places [15]. The attitudes and health seeking behavior towards rabies prevention was found to be 74%. The willingness to register pets was expressed by all the respondents and this is consistent with a study conducted in Sri Lanka. More than 84% responded as being annoyed by the presence of stray dogs, 73.7% support euthanizing stray dogs and more than 87.5% said they would report to the appropriate authority when bitten by a dog. These findings contrast with those in a study in Tanzania which reported that the dog in question would be killed to avert further attack on others [16]. However, a similar study reported the same findings [17]. The correct use of first-aid measures was acceptable to 36.6% of the respondents as recommended by WHO [2] to thoroughly flush wounds with soap and water immediately after a bite injury and povidone iodine or other antiseptic should be applied when available. This figure which is low is however higher than that reported in a study in Ethiopia [18]. More than 86% of the respondents would seek medical care from a hospital or a doctor after being bitten by a dog. This reflects the level of their knowledge on rabies. Another study in Ethiopia also reports that a high number of people would get treated in a hospital [19]. This however contrast to a survey in India where 42% preferred a household treatment such as chili application [20]. Of the total number of respondents, only 55% owned dogs and these were the only ones that responded to the practice section of the KAP. There was 75% satisfactory practice among dog owners. This is reflected in the measures taken to prevent the spread of rabies. Sixty five percent practiced housing dogs in kennels but would allow them to freely roam at some point. In this case, dogs would only be allowed out of their kennels in the night to provide some sought of security while they remained restricted in the daytime to limit direct contact with humans. This is contrary to the study in Addis Ababa [21] and Sri Lanka [22]. Only 9.8% would allow their dogs to roam freely without restriction. The practice of allowing dogs to freely roam would facilitate the spread of rabies in human and animal population. Good practice and responsible dog ownership is also exhibited by the number of those who vaccinated their dogs against rabies (94.3%) and other diseases (94.3%). The rabies vaccination figure is similar to another study conducted in Ontario [23] but contrary to a study in Ethiopia with a very low vaccination rate of 9% [20]. WHO recommends that 80% of total dog population should receive vaccination to give protection against rabies thereby curbing the spread its spread [24]. Dog ownership was statistically significant with knowledge. Dog owners were eight times more likely to have satisfactory knowledge on rabies than non-dog owners. This finding gives credence to the fact that good number of dog owners would vaccinate their dogs against rabies and other diseases as reported in this study. Marital status had significant association with attitudes towards rabies prevention. The non-married were about three time more likely to have positive attitudes towards rabies prevention than the married. Respondents who owned dogs were more likely to be annoyed by stray dogs than non-dog owners. Dog owners would be more knowledgeable about the dangers and health implications posed by roaming dogs and this may have informed this attitude towards roaming dogs.

## Conclusion

Our study showed that knowledge, attitudes and practices with regards to rabies prevention among the residence of AMAC were high. The knowledge and practices of most respondents, irrespective of dog ownership status, were satisfactory with majority also having positive attitudes. The practice of responsible dog ownership was high with majority of the dog owners having their dogs vaccinated against rabies and other vaccine preventable diseases. Rabies vaccine coverage in this study is higher than the recommended WHO coverage of 80% for herd immunity to be achieved. Despite the above, some gaps were observed. They include; not knowing that cure for rabies is not available after symptoms appear, lack of awareness on the first aid measures to be taken after a case of dog bite and danger posed by allowing dogs to stray. Sensitization and awareness campaign to the residents of AMAC is highly recommended to cover these identified gaps to curb the spread and menace of rabies.

### What is known about this topic

- Rabies is a viral zoonoses affecting man, domestic and wild animals;
- Mortality of rabies is highest in Asia and Africa;
- Major host of transmission to man and other animals in Nigeria is the dog.

### What this study adds

- The knowledge of rabies among residents of Abuja Municipal Area Council (AMAC) is very high;
- The need to create more awareness on actions to take towards rabies prevention such as the first aid measures to be taken after a dog bite and that the fact that rabies is not curable after symptoms have appeared because of the knowledge gap in these areas that was observed among respondents;
- Rabies vaccination coverage among dog owners is higher than that recommended by World Health Organization (WHO).

## Competing interests

The authors declare no competing interest.
